# Conjugation of Silver Nanoparticles With De Novo–Engineered Cationic Antimicrobial Peptides: Exploratory Proposal

**DOI:** 10.2196/28307

**Published:** 2021-12-08

**Authors:** Alvin Hu

**Affiliations:** 1 Internal Medicine Residency Indiana University Health Ball Memorial Hospital Muncie, IN United States

**Keywords:** antimicrobial peptides, silver nanoparticles, ESKAPE pathogens, research proposal

## Abstract

**Background:**

Cationic antimicrobial peptides have broad antimicrobial activity and provide a novel way of targeting multidrug-resistant bacteria in the era of increasing antimicrobial resistance. Current developments show positive prospects for antimicrobial peptides and silver nanoparticles (AgNPs) individually.

**Objective:**

The primary objective is to propose another method for enhancing antimicrobial activity by conjugating AgNPs with cationic antimicrobial peptides, with a subsequent preliminary assessment of the minimum inhibitory concentration of multidrug-resistant bacteria. The secondary objective is to evaluate the safety of the conjugated compound and assess its viability for in vivo use.

**Methods:**

The proposal involves 3 stages. First, WLBU2C, a modified version of the antimicrobial peptide WLBU2 with an added cysteine group, needs to be synthesized using a standard Fmoc procedure. It can then be stably conjugated with AgNPs ideally through photochemical means. Second, the WLBU2C-AgNP conjugate should be tested for antimicrobial activity according to the Clinical & Laboratory Standards Institute manual on standard minimum inhibitory concentration testing. Third, the cytotoxicity of the conjugate should be tested using cell lysis assays if the above stages are completed.

**Results:**

I-TASSER (iterative threading assembly refinement) simulation revealed that the modified peptide WLBU2C has a secondary structure similar to that of the original WLBU2 peptide. No other results have been obtained at this time.

**Conclusions:**

The addition of AgNPs to already developed de novo–engineered antimicrobial peptides provides an opportunity for the development of potent antimicrobials. Future prospects include emergency last-line therapy and treatment for current difficult-to-eradicate bacterial colonization, such as in cystic fibrosis, implantable medical devices, cancer, and immunotherapy. As I do not anticipate funding at this time, I hope this proposal provides inspiration to other researchers.

**International Registered Report Identifier (IRRID):**

PRR1-10.2196/28307

## Introduction

There is no longer doubt that present day bacteria are developing increasing resistance to our currently available group of antimicrobial agents [1]. Extensive research has already been performed in the hopes of creating more strategies to counter the increasing resistance of bacteria [1-4]. One such line of study is on cationic antimicrobial peptides (CAPs). CAPs are ubiquitous in nature, being present in all living species [5]. In antimicrobial studies, CAPs are of interest due to their broad antimicrobial spectrum and cellular selectivity [6]. Their novel mechanisms of action, including both membrane and cellular interactions, provide good prospects for drug development against resistant bacteria in a variety of applications [6,7]. WLBU2 is a de novo CAP engineered thematically from the study of naturally occurring CAPs and virally derived peptides called lentivirus lytic peptides from human immunodeficiency virus type 1 [8,9]. It was demonstrated that WLBU2 exerts great antimicrobial activity with the ability to inhibit the growth of multidrug-resistant bacteria, while being safe for mammalian cells [8]. In tandem, the element silver has also been found to have antimicrobial properties. It has practical applications in many health care technologies globally at present, and it is being increasingly studied for its potent antimicrobial and antibiofilm activity [10]. Silver affects bacterial cells by way of membrane disruption and disruption of internal cell processes, similar to CAPs (multiple sources as cited in the report by Franci et al [10]). Given that there are many similarities between WLBU2 and silver nanoparticles (AgNPs), including activity against biofilms and multidrug-resistant bacteria, I propose the conjugation of WLBU2 and AgNP via an additional cysteine amino acid group [1,11,12]. It has been shown that conjugation of AgNPs with proteins has the possibility to negate the negative side effects of both components while retaining the beneficial effects [8,13,14]. This proposal hopes to evaluate the synergistic benefit of AgNPs and WLBU2, and provide points of thought and consideration for future researchers who may find this article useful.

## Methods

### Synthesis of WLBU2C

**Figure 1:**
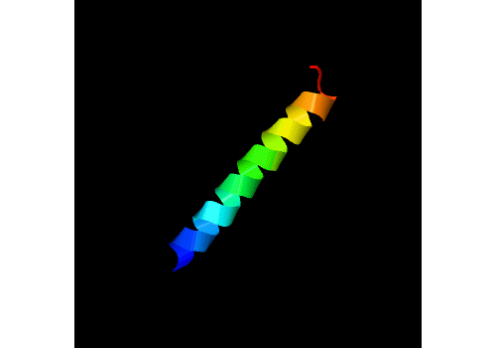
Three-dimensional model from I-TASSER (iterative threading assembly refinement) simulation of the secondary structure of the WLBU2C peptide.

**Figure 2:**
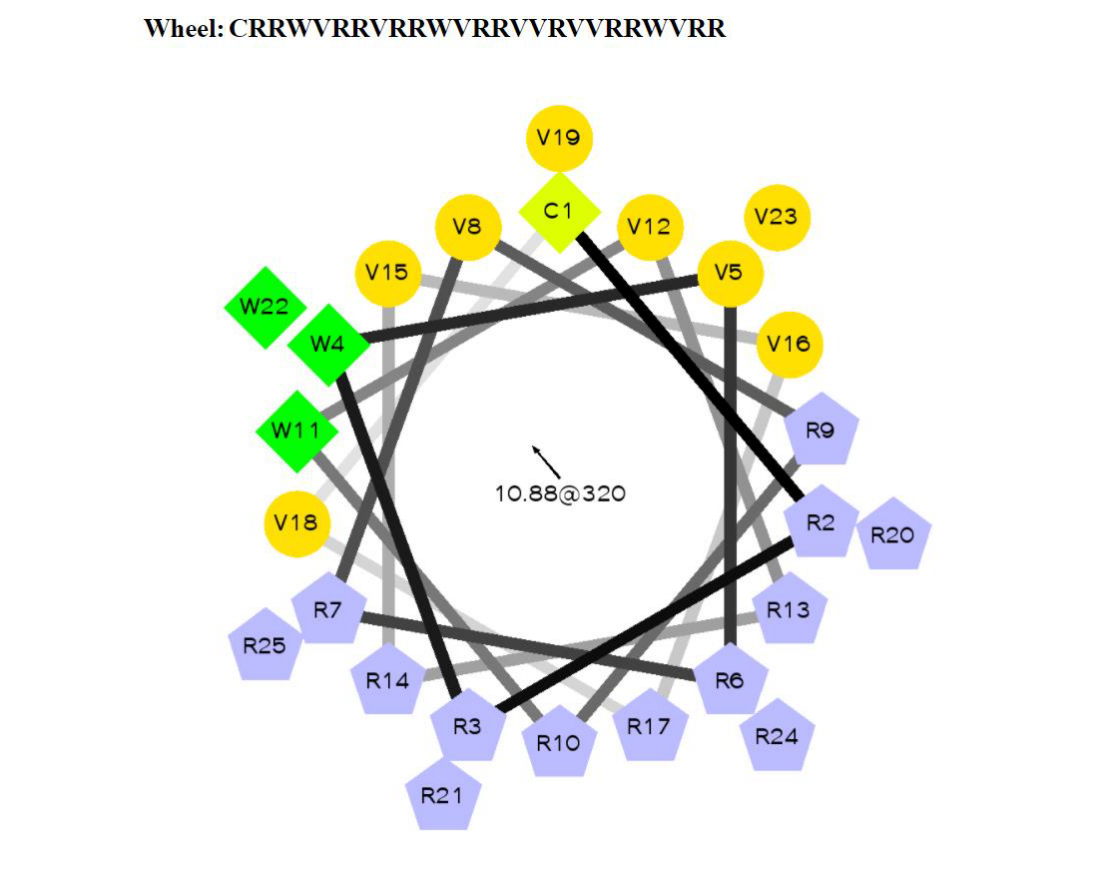
Alpha helical diagram of the proposed WLBU2C peptide shows retained amphipathicity.

This study proposes the synthesis of the conjugate WLBU2-AgNP starting with a modified version of WLBU2 (hereafter called WLBU2C) (wheel: CRRWVRRVRRWVRRVVRVVRRWVRR). The development of this peptide is theoretically proposed by assessing the structure of WLBU2C through I-TASSER (iterative threading assembly refinement) simulation and an alpha helical diagram (Figure 1 and Figure 2) to retain amphipathicity and secondary structure [15-17]. Synthesis should be performed with the standard Fmoc procedure, after which the product should be purified with reversed-phase high-performance liquid chromatography using a C18 Vydac column as the stationary phase and be confirmed with mass spectrometry. The secondary structure can be evaluated with circular dichroism (CD) in the presence of phosphate buffer with saline (PBS) for an aqueous environment or 30% trifluoroethanol for a membrane mimetic environment [8]. The addition of cysteine as the conjugation point between the proposed antimicrobial peptide and AgNP has been theorized through review of prior studies in which cysteine group conjugation provided enhanced binding and stability with increased activity against *Klebsiella pneumoniae* [18,19].

### Synthesis of WLBU2C-AgNP

This study proposes the synthesis of WLBU2C conjugated to AgNP (hereafter called WLBU2C-AgNP) by means of a photochemical method, as similarly described for LL37@AgNP by Vignoni et al [14]. The method specifically adapted for this research involves the use of deoxygenated silver nitrate (AgNO_3_), Igracure 2959 as a photo initiator, and WLBU2C in sliding scale concentrations from 0 to 100 μM to test for the optimal concentration [20]. UVA lamps equivalent to Luzchem CCPV4 photoreactors should be used at 25°C, and the reaction can be monitored with UV-visible absorption spectroscopy. Based on the literature, the surface plasmon band is expected to be centered at around a wavelength of 395 to 425 nm [14]. According to a review of the above studies, it is expected that the absorption will increase in the UV-visible spectrum until all the Ag^+^ molecules are reduced.

After synthesis and purification with a dialyzer of appropriate size, transmission electron microscopy and dynamic light scattering (DLS) should confirm the presence of a larger DLS particle size due to the binding of the proteins around the nanoparticles. The secondary structure can be evaluated by CD, unless the nanoparticles interfere with CD resolution.

### Antibacterial Activity Evaluation

Testing of antibacterial activity is suggested against ESKAPE pathogens (*Enterococcus faecium*, *Staphylococcus aureus*, *Klebsiella pneumoniae*, *Acinetobacter baumannii*, *Pseudomonas aeruginosa*, and *Enterobacter* species). Bacterial killing can be evaluated in the setting of potassium phosphate buffer and PBS by the dilutional assay method, in which WLBU2C-AgNP (0-100 μM) is mixed with bacteria (diluted to 1×10^6^ colony forming units/mL) at 37°C for 60 minutes, and the mixture is then plated and incubated (appropriate conditions and time) [8]. Subsequent analysis may be performed with spectrophotometry at 600 nm [21]. Further information regarding the tests for the minimum inhibitory concentration and minimum bactericidal concentration can be obtained from the Clinical & Laboratory Standards Institute manual for antibacterial susceptibility testing.

### Cytotoxicity Evaluation

Once antimicrobial activity has been assessed, cytotoxicity can be evaluated against human red blood cells and normal cells, such as keratinocytes and fibroblasts, to explore practicality. The cytotoxicity protocol can be derived from the procedures performed by Deslouches et al [22]. Briefly, a red blood cell hemolytic assay can be performed in PBS by changing the WLBU2C-AgNP concentration. Further cytotoxicity can be assessed by culturing keratinocytes and fibroblasts in Dulbecco Eagle’s medium and performing tests with a range of WLBU2C-AgNP concentrations, as well as an MTT assay for metabolic activity.

## Results

This proposal is currently theoretical and does not have reportable results other than structure simulations as listed above, where it was found that the modified WLBU2C peptide has a secondary structure similar to that of the original WLBU2 peptide. I do not anticipate proactively obtaining funding in the future due to insufficient resources.

## Discussion

### Limitations

Anticipated limitations of this study include the short half-life of CAPs and associated cytotoxicity in higher concentrations, which may be counteracted with immobilization of the peptide onto solid surfaces [23]. Silver might be toxic to mammalian cells and the environment [10]. As all proposed approaches are theoretical, there is no guarantee to achieve the expected outcome.

### Conclusions

Recently, more research has been reported regarding the combination of antimicrobial peptides with AgNPs, with positive results [18,24]. This proposal provides another idea to efforts for counteracting antimicrobial resistance. It has been hypothesized that the conjugation of a de novo–engineered antimicrobial peptide and AgNP may increase the antibiofilm effect against multidrug-resistant bacteria while retaining selectivity and safety. The present method involving cysteine group modification on the antimicrobial peptide for conjugation with AgNP has to my knowledge not yet been published for de novo–engineered CAPs. De novo–engineered antimicrobial peptides are still undergoing active research to increase their potency while balancing cytotoxicity. The conjugation of improved peptides with AgNPs would provide a second degree of freedom to their functions, hopefully unlocking opportunities to develop more potent antimicrobials. If further studies on this topic are successful, future long-term prospects may include emergency last-line antibiotic therapy and treatment for difficult-to-eradicate bacterial colonization, such as in cystic fibrosis, implantable medical devices, cancer, and immunotherapy [25]. I encourage further studies on this topic to better understand the proposed theories. As I do not anticipate proactively obtaining funding for this idea in the future, I hope this proposal provides inspiration to other researchers.

## Abbreviations

AgNP: silver nanoparticle
CAP: cationic antimicrobial peptide
CD: circular dichroism
DLS: dynamic light scattering
PBS: phosphate buffer with saline
